# In Situ Exfoliation Method of Large‐Area 2D Materials

**DOI:** 10.1002/advs.202301243

**Published:** 2023-05-26

**Authors:** Antonija Grubišić‐Čabo, Matteo Michiardi, Charlotte E. Sanders, Marco Bianchi, Davide Curcio, Dibya Phuyal, Magnus H. Berntsen, Qinda Guo, Maciej Dendzik

**Affiliations:** ^1^ Zernike Institute for Advanced Materials University of Groningen Groningen 9747 AG The Netherlands; ^2^ Department of Applied Physics KTH Royal Institute of Technology Hannes Alfvéns väg 12 Stockholm 114 19 Sweden; ^3^ Quantum Matter Institute University of British Columbia Vancouver BC V6T 1Z4 Canada; ^4^ Department of Physics and Astronomy University of British Columbia Vancouver BC V6T 1Z1 Canada; ^5^ Central Laser Facility Research Complex at Harwell Rutherford Appleton Laboratory Harwell Campus Didcot 0X11 0QX UK; ^6^ School of Physics and Astronomy Aarhus University Aarhus 8000 C Denmark

**Keywords:** 2D materials, angle‐resolved photoemission spectroscopy, band structure, exfoliation, transition metal dichalcogenides

## Abstract

2D materials provide a rich platform to study novel physical phenomena arising from quantum confinement of charge carriers. Many of these phenomena are discovered by surface sensitive techniques, such as photoemission spectroscopy, that work in ultra‐high vacuum (UHV). Success in experimental studies of 2D materials, however, inherently relies on producing adsorbate‐free, large‐area, high‐quality samples. The method that yields 2D materials of highest quality is mechanical exfoliation from bulk‐grown samples. However, as this technique is traditionally performed in a dedicated environment, the transfer of samples into vacuum requires surface cleaning that might diminish the quality of the samples. In this article, a simple method for in situ exfoliation directly in UHV is reported, which yields large‐area, single‐layered films. Multiple metallic and semiconducting transition metal dichalcogenides are exfoliated in situ onto Au, Ag, and Ge. The exfoliated flakes are found to be of sub‐millimeter size with excellent crystallinity and purity, as supported by angle‐resolved photoemission spectroscopy, atomic force microscopy, and low‐energy electron diffraction. The approach is well‐suited for air‐sensitive 2D materials, enabling the study of a new suite of electronic properties. In addition, the exfoliation of surface alloys and the possibility of controlling the substrate‐2D material twist angle is demonstrated.

## Graphical abstract

2D materials provide a rich platform to study novel physical phenomena arising from quantum confinement. This article presents a simple and generic method of kinetic in situ single‐layer synthesis, which enables the exfoliation of sub‐millimeter flakes of air‐sensitive 2D materials directly in vacuum. The method does not require the usage of a glovebox or other specialized equipment, making it well‐suited for surface science techniques.

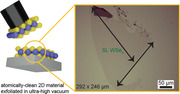

## 1 Introduction

The era of 2D materials began with the discovery of graphene^[^ ^1^ ^]^– a single layer of graphite, which exhibits outstanding physical properties that do not exist in its bulk counterpart.^[^ ^2^, ^3^, ^4^, ^5^, ^6^, ^7^ ^]^ The research that followed quickly expanded into other types of 2D materials, such as transition metal dichalcogenides (TMDCs),^[^ ^8^, ^9^, ^10^, ^11^, ^12^, ^13^, ^14^, ^15^ ^]^ hexagonal boron nitride,^[^ ^16^, ^17^ ^]^ and MX‐enes^[^ ^18^, ^19^ ^]^ to name a few. Among these, TMDCs are particularly interesting as semiconducting single layer (SL) TMDCs possess a direct band gap in the visible range,^[^ ^10^, ^20^ ^]^ which makes them exceptional candidates for opto‐electronic devices,^[^ ^21^, ^22^ ^]^ and metallic SL TMDCs showcase properties such as 2D Mott physics, superconductivity, and topological phases.^[^ ^23^ ^]^ Furthermore, many physical properties of 2D materials are sensitive to careful interface engineering through the stacking of layers into homo‐ and heterostructures as well as the adjustment of the twist angle between adjacent layers.^[^ ^24^, ^25^, ^26^, ^27^, ^28^ ^]^ To effectively study and work with 2D materials, the research field relies heavily on efficient methods of producing high‐quality samples. The most significant challenges are posed by the strict requirements imposed on the sample crystallinity, atomic‐level cleanliness of the interface, and sample size, which must be larger than the size of the (experimental) probe.

One way to produce 2D materials is to employ well‐established techniques such as chemical vapor deposition (CVD) and molecular beam epitaxy (MBE) to grow ultra‐thin films in situ.^[^ ^11^, ^14^, ^29^, ^30^, ^31^, ^32^, ^33^ ^]^ The major drawbacks of epitaxial techniques are the complexity of determining new stable growth recipes, the choice of an appropriate substrate, and the sample mosaicity, which pose challenges that are unique for each material and make it difficult to generate large samples with good crystallinity. On the other hand, mechanical exfoliation of 2D materials does not encounter the same obstacles, as the material is first grown in its bulk form and later exfoliated to the 2D limit, thus producing flakes of the highest quality.^[^ ^34^, ^35^ ^]^ Although conceptually simple, exfoliation usually suffers from low yield of flakes, which are small in size,^[^ ^34^, ^36^ ^]^ stochastic distribution of the thickness, and residual contamination.^[^ ^37^, ^38^ ^]^ As such, exfoliation produces samples with good crystallinity, but struggles with sample size and contamination. Since the commonly used sample synthesis methods all have severe drawbacks, finding a solution to these problems is highly desirable for research in the field. Recently, some pioneering work has shown that large‐area flakes of TMDCs can be deposited onto freshly‐evaporated polycrystalline gold substrates by exploiting the strong chemical affinity between the metal and the sample.^[^ ^39^, ^40^, ^41^, ^42^, ^43^ ^]^ This approach, however, often requires specialized equipment and the whole procedure can be time‐consuming and difficult to perform.

Here, we propose a new, simple, and generic method to efficiently exfoliate ultra‐clean and large‐area 2D materials: kinetic in situ single‐layer synthesis (KISS). We show that exfoliation of 2D materials can be performed in ultra‐high vacuum (UHV) using various substrate materials, unlike previous works that were limited to the Au substrate under ambient conditions.^[^ ^39^, ^40^, ^41^ ^]^ The general idea of the KISS method is to bring the atomically‐clean surfaces of the bulk layered material and the substrate into contact inside a vacuum environment, thus establishing a bonding interaction that facilitates the exfoliation of single layers. We show that the KISS technique produces sub‐millimeter flakes with excellent quality and purity when exfoliating using both metallic and semiconducting substrates (Au(111), Ag(111), and Ge(100)). Like any exfoliation technique, the substrate‐2D material twist angle is arbitrary and not constrained by epitaxy rules. This provides an opportunity for interface engineering of 2D‐heterostructures, provided that the alignment of the van der Waals material and substrate is selected before the KISS exfoliation. We employ angle‐resolved photoemission spectroscopy (ARPES), low‐energy electron diffraction (LEED), atomic force microscopy (AFM), and optical microscopy to assess the sample quality and properties. This exfoliation procedure does not require specialized equipment the beyond standard UHV apparatus, and further surface‐sensitive experiments are performed without any post‐synthesis cleaning treatment.

## 2 Results and Discussion

### 2.1 Description of the KISS Method

**Figure 1:**
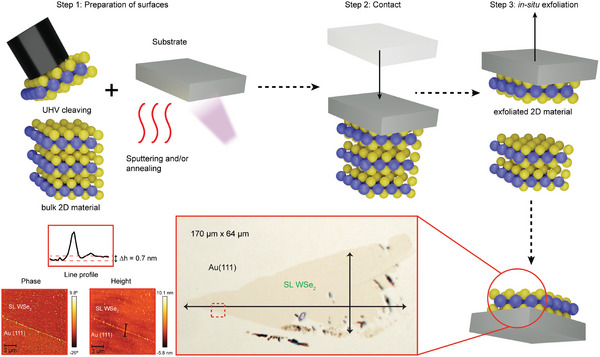
Sketch of the KISS exfoliation procedure. In the step 1, the sample surface is cleaved in UHV to expose an adsorbate‐free surface and the single‐crystal metal substrate is sputtered and annealed to generate an atomically‐clean and flat surface. In the step 2, the two surfaces are brought into contact. In the step 3, the sample and substrate are gently separated, resulting in the in situ exfoliation of the 2D material onto the substrate. A high‐quality, large‐area 2D material is left on the substrate, as seen in the optical microscopy image for the case of SL WSe_2_ on Au(111). The SL thickness of the sample is demonstrated by its characteristic height of 0.7 nm as measured by AFM data in the region marked with a red dashed square in the optical image.

**Figure** **1** presents an overview of the KISS method. First, atomically‐clean surfaces are prepared on both the substrate and the bulk 2D material using standard techniques directly in UHV; the substrate is prepared via cycles of ion sputtering and annealing, and the bulk 2D material is cleaved in situ. In the second step, the two surfaces are slowly brought into contact to establish a strong bonding interaction between the 2D material and the substrate. In the last step, the materials are slowly and rigidly separated resulting in the in situ exfoliation of the 2D material. The obtained flakes of SL WSe_2_ on Au(111) are routinely hundreds of micrometers in size (see optical microscope image shown in Figure 1) and of excellent structural quality, as confirmed by LEED (Figure S1, Supporting Information). To determine the thickness of the flake, we measure its height with AFM as shown in Figure 1 and Figure S2, Supporting Information. The obtained value of ≈0.7 nm is in agreement with previously reported values for SL WSe_2_.^[^ ^44^, ^45^ ^]^

### 2.2 Exfoliation of Large‐Area SL WSe_2_ on Ag(111)

**Figure 2:**
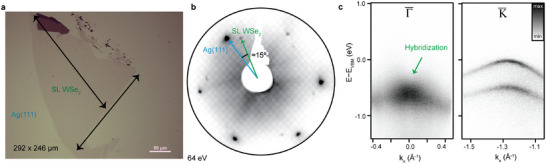
Silver‐assisted exfoliation of large‐area SL WSe_2_. a) The optical microscopy image shows a flake of SL WSe_2_ exfoliated by the KISS method onto Ag(111); the two major dimensions indicated by arrows are 292 µm and 246 µm. The color balance was adjusted to make the single layer more visible. The dark region near the top is a small multi‐layered region. b) LEED image of the WSe_2_ flake; the angle between the Bragg peaks of the sample and the substrate is ≈15°, confirming that the exfoliation angle can be arbitrarily chosen and does not rely on coherent epitaxy. c) ARPES data showing WSe_2_ bands around the $\bar{\Gamma }$ (left) and $\bar{K}$ points (right). The energy axis is referenced to the valence band maximum (VBM) that is located at the $\bar{K}$ point. The green arrow indicates where the hybridization between the WSe_2_ and Ag(111) bands appear. The higher energy of the bands at the $\bar{K}$ point is characteristic of the SL nature of the flake.

As a test case for the KISS method, SL WSe_2_ was also exfoliated onto Ag(111) and characterized in detail. A WSe_2_ bulk crystal and Ag(111) substrate were prepared as described in Experimental Section, and the exfoliation procedure was carried out as described above. The KISS‐exfoliation resulted in a large‐area SL WSe_2_ flake of 292 µm × 246 µm, shown in **Figure** **2**a. The SL flake is continuous and homogeneous, with smaller multi‐layer regions close to the edges (darker color in the optical microscope image). LEED data confirm the single domain crystallinity of the exfoliated flake, as only a single set of the WSe_2_ diffraction spots can be seen in Figure 2b. The WSe_2_ flake is rotated by ≈15° with respect to the underlying Ag(111) substrate, as indicated by the blue and green arrows.

We performed ARPES measurements to further evaluate the sample thickness and quality (see Figure 2c). It must be noted that because the KISS technique is performed in UHV, the exfoliation happens in situ, and samples are transferred to the ARPES chamber without being exposed to any gas or pollutants. Overall, the ARPES data around the $\bar{K}$ point is of exceptionally high quality for exfoliated flakes of 2D materials, indicating the high level of crystallinity, purity, and flatness. As expected for a SL, the valence band maximum (VBM) is located at the $\bar{K}$ point, shown in Figure 2c (right).^[^ ^46^, ^47^ ^]^ The observed large spin‐orbit splitting of (473±7) meV, linewidths of (53±7) meV (upper VB), and (104±7) meV (lower VB) at the $\bar{K}$ point, and effective hole mass of (0.54±0.06) *m*_0_, are in good agreement with previously reported values.^[^ ^47^, ^48^ ^]^ A single valence band is found at the Brillouin zone center, the $\bar{\Gamma }$ point, shown in Figure 2c (left), confirming that the exfoliated material is indeed a SL.^[^ ^46^, ^47^ ^]^ One can learn a lot about the physical interaction between the substrate and the WSe_2_ flake from a careful inspection of its electron dispersion: hybridization of the 2D material bands with the substrate indicates a strong bonding interaction, which manifests as broader and gapped spectral features. The valence band of WSe_2_ at the $\bar{K}$ point is mostly derived from in‐plane orbitals and does not hybridize with the substrate, resulting in the sharp bands observed. Contrarily, the valence band at the $\bar{\Gamma }$ point primarily derives from out‐of‐plane chalcogen p_*z*_ and transition metal $d_{z^{2}}$ orbitals, which are directly involved in the bond with the substrate.^[^ ^49^ ^]^ As evident in Figure 2c, the band is significantly broader at the $\bar{\Gamma }$ point where electron bands from Ag(111) are crossing the WSe_2_ bands, causing hybridization, which indicates that a strong bonding interaction has been established.^[^ ^50^ ^]^ This TMDC‐substrate interaction is stronger than the inter‐layer interaction holding bulk TMDCs together, and it is responsible for the exfoliation of single layers. It is important to note that despite the chemisorption to the substrate, we did not observe any preferential rotational alignments between TMDC flakes and the Ag(111) or Au(111) substrates. This is consistent with the radial symmetry of the out‐of‐plane orbitals participating in the bond. The natural implication is that the orientation of the flake on the substrate is arbitrary during the KISS, and could provide a way to choose the “twist” angle at the interface, if both angles are characterized and determined ahead of the KISS exfoliation.

Overall, TMDC exfoliation onto the surface of silver seems to yield more copious and larger flakes than onto gold, likely because of a stronger sample/substrate interaction.^[^ ^50^ ^]^ However, it is important to note that the silver surface passivates more quickly when exposed to air, and a similar exfoliation process performed outside vacuum conditions might be impaired by this process.

### 2.3 Survey of Suitable Substrates and TMDC Materials

**Figure 3:**
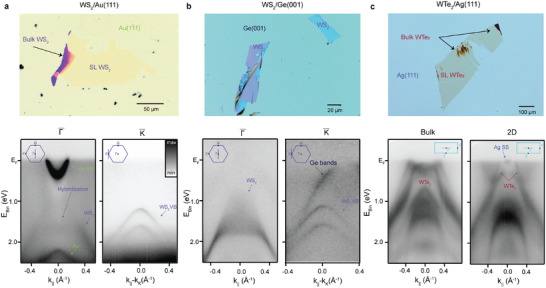
Universality of the KISS method: Exfoliation of different TMDC materials onto multiple substrates. Optical microscopy images are shown in the top row and ARPES data acquired in situ in the bottom row for three different systems. The insets illustrate the cuts in momentum space that are plotted in the respective ARPES spectra. a) Flakes of WS_2_/Au(111) are over 100 µm in size and predominantly SL as demonstrated by the photoemission data, where the valence band maximum is at the $\bar{K}$ point. Above the $\bar{\Gamma }$ point, an Au(111) surface state is visible. b) Exfoliated flakes of WS_2_/Ge(001) demonstrate that exfoliation of 2D materials directly onto semiconducting substrates is also possible. The ARPES data is of high quality, but the flakes are generally smaller and thicker than on metallic substrates. c) 2D WTe_2_ has been exfoliated onto Ag(111), yielding flakes of hundreds of microns in size. The KISS exfoliation method is particularly useful for air‐sensitive samples, such as WTe_2_, which can be exfoliated and measured in situ. The ARPES data is of remarkable quality and is shown from both a multilayered (3D) area of the flake (left) and from a 2D area (right). The spectra are markedly different, especially close to the Fermi level, highlighting the vast topological difference between the two cases.

We have explored the KISS method of exfoliation across various van der Waals layered materials and substrates. **Figure** **3** presents results for the TMDCs WTe_2_ and WS_2_, on the substrates Au(111), Ag(111), and Ge(001). As shown in Figure 3a the exfoliation of WS_2_ yields similar results to WSe_2_ with a large SL flake and well‐defined spectral features. A valence band spin‐splitting of ≈430 meV can be seen at the $\bar{K}$ point, which is in agreement with the literature.^[^ ^51^, ^52^ ^]^ An Au(111) surface state^[^ ^53^ ^]^ is still clearly visible at the $\bar{\Gamma }$ point. The strong signal indicates that the surface of the substrate was not damaged or contaminated during the KISS exfoliation. Additional measurements for MoS_2_ exfoliated onto Ag(111) can be found in Figure S3, Supporting Information.

Up to this point, we have only considered noble metal substrates. However, the surfaces of semiconductors prepared in UHV often host dangling bonds, which can facilitate the adherence of the 2D material in a similar fashion as seen for metals. Insulating and semiconducting substrates enable access to the transport properties of pristine 2D materials, and the use of semiconducting substrates is, therefore, very desirable. Figure 3b shows the results of KISS exfoliation of WS_2_ onto the (001) surface of germanium. Similar to the exfoliations using Ag(111) and Au(111), thin flakes of 2D WS_2_ are observed on the surface. However, the average size of the flakes seems to be smaller, with a higher prevalence of multilayer regions. Altogether, these results support the breadth of the applicability of the KISS method, indicating the availability of a broad potential pool of suitable semiconducting or metallic substrates with different surface orientations, which likely goes beyond what was tested in this work.

Perhaps the most interesting application of the KISS method is the exfoliation of metallic 2D materials. These systems exhibit fascinating electronic properties in their bulk forms that have been subject to decades of studies, such as the superconductivity in NbSe_2_,^[^ ^54^ ^]^ the remarkable charge density wave phases of TaSe_2_,^[^ ^55^, ^56^, ^57^ ^]^ or the Weyl semimetal state of WTe_2_.^[^ ^15^, ^58^ ^]^ In most cases, these materials cannot be exfoliated in air due to their debilitating air sensitivity, causing their surface to degrade outside of vacuum or an inert atmosphere. This technical inconvenience poses a big challenge in studying their 2D counterparts, as the exfoliated material must be kept and transferred in an inert atmosphere at all times. The KISS exfoliation is ideal for these materials, as it can be performed in UHV, directly in the experimental setup. We study the case of WTe_2_ to prove this applicability.

The exfoliation of WTe_2_ flakes is generally afflicted not only by air sensitivity, but also by low yield of small flakes, on the scale of a few micrometers in size.^[^ ^58^, ^59^, ^60^ ^]^ Figure 3c presents the results of WTe_2_ exfoliated onto Ag(111) using the KISS method, which produced very large flakes of predominantly SL thickness. ARPES data were acquired from regions with bulk (Figure 3c (left)) and 2D (Figure 3c (right)) character. In contrast to the previously reported 2D WTe_2_ grown on bilayer graphene, where three rotational domains are present due to the weak substrate interaction and threefold symmetry of the substrate,^[^ ^15^ ^]^ only a single domain of WTe_2_ is obtained using the KISS method (see Figure S4, Supporting Information). We also note that the WTe_2_ presented here appears to be n‐doped, likely due to charge transfer from the underlying Ag(111). The flakes exfoliated by the KISS technique are hundreds of microns large, demonstrating that this approach allows for in situ study of large‐area flakes of metallic 2D materials.

### 2.4 2D Materials Beyond TMDCs

Unlike TMDCs or graphene, many 2D systems, such as silicene,^[^ ^61^, ^62^ ^]^ germanene,^[^ ^63^, ^64^ ^]^ bismuthene,^[^ ^65^, ^66^ ^]^ or surface alloys, are stabilized by their interaction with the substrate. This presents significant difficulties in the synthesis of some of these materials. Previous successful attempts were realized exclusively via various CVD and MBE growth methods by careful tuning of the substrate‐adlayer interaction via controlling of the physical conditions such as temperature. Exfoliation of 2D materials at elevated temperatures inside a glovebox is challenging due to rapid oxidation and degassing of contamination. KISS exfoliation, on the other hand, provides a unique way of achieving such a control while circumventing the above problems due to the UHV environment. We have followed this idea by investigating surface alloying process, which is a recognized route toward engineering unique physical and chemical properties not found in the bulk materials.^[^ ^9^, ^67^, ^68^, ^69^ ^]^ These include the appearance of Dirac nodal line fermions in CuSe grown on Cu(111),^[^ ^70^ ^]^ and a giant Rashba‐type spin splitting in Ag_2_Bi on Ag(111)^[^ ^71^, ^72^ ^]^ and AgTe on Ag(111),^[^ ^73^, ^74^ ^]^ which shows the presence of an orbitally‐driven Rashba effect.^[^ ^75^ ^]^

**Figure 4:**
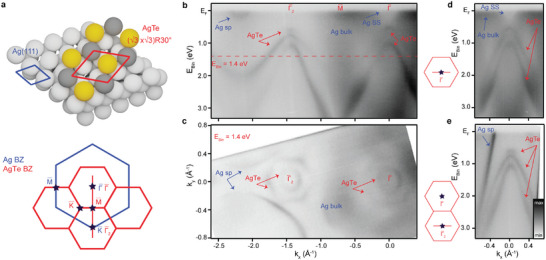
Formation of AgTe alloy on Ag(111). a) Model of the crystal structure of the AgTe alloy on Ag(111) (top) and sketch of the corresponding surface Brillouin zones (bottom). b) AgTe band structure along the $\bar{\Gamma }$–$\bar{K}$ direction of the substrate. $\bar{\Gamma }$, $\bar{M}$, and ${\bar{\Gamma }}_{2}$ mark the high‐symmetry points of AgTe. The dashed red line indicates where the constant energy contour in (c) is taken. c) Constant energy contour taken at E_Bin_ = 1.4 eV. Bulk and surface states (SS) of Ag(111) are visible, together with the AgTe bands. d,e) The AgTe band structure taken at the $\bar{\Gamma }$ point in the first surface Brillouin zone and the ${\bar{\Gamma }}_{2}$ point in the second surface Brillouin zone, respectively. The directions of the cuts directions are indicated in the schematics next to the data. Blue and red arrows indicate Ag and AgTe bands, respectively.

Hexagonal 2D AgTe was synthesized by KISS exfoliation by approaching the Te‐rich surface of WTe_2_ to Ag(111) at a temperature of 373 K. The higher procedural temperature enables the mobile Te atoms to self‐organize on the Ag(111) surface into the ($\sqrt{3}\times \sqrt{3}$)R30° superstructure depicted in **Figure** **4**a. After the exfoliation, we found regions containing amorphous Te clusters and large areas of high‐quality AgTe on the substrate surface. The symmetry of the AgTe alloy and its electronic structure (Figure 4b–e) are in excellent agreement with the results reported in refs. [73, 74, 75] for samples grown with epitaxial techniques. The presence of bands related to the AgTe layer is evident in Figure 4b, which shows a cut of the ARPES data along the $\bar{\Gamma }$–$\bar{K}$ high‐symmetry direction of the Ag(111) lattice. The constant energy contour at a binding energy of 1.4 eV (Figure 4c) reveals Ag(111) bulk and surface states (blue arrows), and threefold symmetric AgTe states (red arrows) around the $\bar{\Gamma }$ and $\bar{K}$ points of the surface Brillouin zone (SBZ) of the Ag(111) substrate. The observed dispersion of the AgTe states around these points is found to be identical (Figure 4d,e), which is consistent with the proposed ($\sqrt{3}\times \sqrt{3}$)R30° periodicity shown in the Figure 4a. The exact structure of the obtained AgTe is deduced by comparison of the measured electronic band structure with the results of density functional theory calculations presented in ref. [73]. High‐quality ARPES data showing threefold symmetry of the observed AgTe bands indicate that the layer is a single domain.

These results bring to light an alternative approach for fast and straightforward synthesis of 2D materials in a manner that does not require either specialized equipment, such as source‐material evaporators, nor the time consuming outgassing and deposition processes necessary for MBE growth.

## 3 Conclusions

Ambient Au‐assisted exfoliation methods^[^ ^39^, ^40^, ^41^ ^]^ have demonstrated that two conditions facilitate the large‐area flake synthesis: 1) substrate cleanness and 2) macroscopic surface roughness. Meeting condition (1) in air or inside glove box environment is problematic because of the requirement for exfoliation to be performed immediately after metallic surface evaporation, and because most ex vacuo surfaces will quickly undergo chemistry or adsorption of impurities, even in a glovebox. Recent work has shown that the Ag substrate exhibits a similar exfoliation yield as Au,^[^ ^76^ ^]^ but the observed efficiency declines rapidly within ≈10 s due to surface oxidation. This effect limits the possible choice of substrate to relatively non‐reactive Au or Ag and makes the exfoliation technically challenging. On the other hand, performing UHV exfoliation using the KISS approach enables in vacuo preparation of a clean metal or semiconductor substrate surface by any of a plethora of available techniques, and drastically increases the time that surface can stay clean. Concerning the condition (2), we performed in‐air AFM investigations of the UHV‐cleaned substrates and optical imaging of UHV‐cleaved 2D materials used in this work (Figures S7 and S8, Supporting Information). We find a typical roughness of the substrates to be in the range of 1–3 nm for areas of several millimeters in size. Optical images of cleaved layered materials reveal a typical domain size of sub‐millimeter which we attribute to be a main limiting factor of KISS‐exfoliated sample size. Once again, as surface roughness can be efficiently decreased by low‐energy grazing angle ion sputtering in UHV,^[^ ^77^ ^]^ the KISS method should be favorable in regard to the condition (2).

The most significant limitation of the KISS exfoliation method originates from the inherent interaction between a substrate and a 2D material. On one hand, substrate interaction is critical for getting adhesion between the flake and the substrate during the KISS process. On the other, substrate‐induced interactions can severely affect some of the most interesting physical properties of van der Waals crystals. For example, it was shown that SrTiO_3_ can increase the superconducting temperature of ultra‐thin FeSe above 100 K.^[^ ^78^ ^]^ It has been shown that even relatively weak hybridization with the substrate can lead to significant changes in the electronic structure of 2D materials,^[^ ^50^ ^]^ including the suppression of correlated many‐body states.^[^ ^79^ ^]^ We expect that samples produced using the KISS method will exhibit similar phenomena. Nevertheless, subsequent methods to lift the substrate interactions to obtain quasi‐free standing 2D materials can be used or further developed. These include, for instance, hydrogen intercalation (as has been demonstrated for graphene on SiC^[^ ^80^ ^]^), oxygen intercalation (as used for graphene on Ir(111)^[^ ^81^ ^]^), or bismuth intercalation (as for WS_2_ on Ag(111)^[^ ^82^ ^]^). The nano‐patterning of substrates is also a further avenue to be explored.

In summary, we show a new method to produce large‐area and atomically‐flat flakes of 2D materials in UHV. This technique is versatile and applicable to a variety of TMDCs and different substrates. The flakes produced are single‐domain and several hundreds of microns in size, enabling direct in situ measurements with most spectroscopy, diffraction, microscopy, and transport techniques. The in‐plane angle between the flake and the substrate can be arbitrarily chosen, thus facilitating the production of twisted heterostructures. Moreover, the possibility of exfoliating in a UHV environment allows for the direct in situ study of the exfoliated material with techniques such as ARPES and scanning tunneling microscopy, even for air‐sensitive samples that otherwise must be either grown in UHV or capped upon preparation in a glove box. We also show the large‐area exfoliation of metallic TMDCs that have so far remained elusive because of their air sensitivity. A compelling aspect of the technique is highlighted by the fact that the presented ARPES data were obtained using two experimental setups with macroscopic beam footprints in contrast to typical ARPES measurements on exfoliated flakes, which require nanofocusing capabilities—a feature only available in a handful of facilities in the world. Our results provide a new platform to study a wide variety of novel systems exhibiting interesting phenomena, such as the topological superconductivity in twisted, high‐temperature superconductor heterostructures^[^ ^83^ ^]^ or the burgeoning field of 2D magnetism.^[^ ^84^ ^]^

## 4 Experimental Section

### Materials Preparation:

Au(111) single crystal (MaTecK), Au(111) thin film on mica (≈200 nm thickness, Phasis), and Ag(111) thin film (≈300 nm thickness, Georg‐Albert‐PVD) on mica were prepared by repeated cycles of Ar^+^ (or Ne^+^) sputtering (5 × 10^−5^ mbar Ar (Ne), 1.7 kV, 30 min), and annealing (30 min at 600 K or 30 min at 800 K, depending on the setup). Ge(001) was prepared by Ar^+^ (Ne^+^) sputtering (3.4 × 10^−6^ mbar Ar (Ne), 1.0 kV, 15 min), and annealing (15 min at 900 K). Bulk TMDC crystals (HQ graphene) were cleaved by the top‐post method at chamber pressure better than 1 × 10^−8^ mbar. The quality of the as‐cleaved vdW crystals was checked prior to KISS exfoliation by ARPES to confirm the cleanliness and expected bulk structure. Bulk Au(111) was used for the KISS exfoliation shown in Figure 1, while Au(111) and Ag(111) on mica were used for the KISS exfoliations shown in Figures 2, 3, 4.

### In situ Exfoliation (the KISS Method):

Samples with freshly cleaned surfaces were brought into direct contact in UHV by manual movement of a manipulator (micrometer screw) or transfer arm, on which they were located. For the KISS exfoliation done at the BALTAZAR facility, the vdW material was kept on a standard sample holder, while at the SGM3 beamline an appropriate spring‐loaded sample plate was used for the vdW material. An example of spring‐loaded samples plates used for the KISS‐exfoliation can be seen in Figure S6, Supporting Information. Following contact between the surfaces, where a 2.45 N force was measured to have been used, the substrate and bulk crystal were brought out of contact by slow movement of the manipulator in the opposite direction. The exfoliated flakes were found on the substrate by the use of ARPES raster‐mapping to locate signals originating from core levels or valence bands. See Figure S5, Supporting Information. The KISS exfoliation was performed at pressures better than 5 × 10^−10^ mbar. The KISS exfoliation was done at room temperature, except in the case of the AgTe alloy, where it was done at an elevated temperature of ≈373 K.

### Low‐Energy Electron Diffraction:

LEED measurements were performed following the KISS exfoliation and taken at room temperature with an ErLEED 150, SPECS system, with a spot size of ≈1 mm.

### Atomic Force Microscopy Characterization:

AFM measurements were performed under ambient conditions with Dimension FastScan, Bruker and Cypher A AFM, Asylum Research instruments. The measurements were performed using tapping mode, following ARPES and LEED measurements. The AFM data was analyzed using Gwyddion software package.^[^ ^85^ ^]^

### Angle‐Resolved Photoemission Spectroscopy:

ARPES measurements were performed at the BALTAZAR laboratory (KTH Royal Institute of Technology in Stockholm, Sweden)^[^ ^86^ ^]^ and at the SGM3 beamline of the synchrotron radiation facility ASTRID2 (Aarhus University, Denmark).^[^ ^87^ ^]^ The measurements were performed at room temperature with base pressure better than 1 × 10^−10^ mbar. At the BALTAZAR facility, a high‐power femtosecond laser (Amplitude, Tangor 100) with an adjustable repetition rate (from a single shot to 40 MHz), providing infrared pulses centered at 1030  nm, was used for high harmonic generation in Ar gas. A repetition rate of 250 kHz, and the fifth harmonic of *h*ν = 18.1 eV, were used for the experiments. The measurements were performed with an ARTOF analyzer (SPECS, Themis 1000), with energy and angular resolutions of 14 meV and 0.1°, respectively. The beam footprint on the sample was ≈100 µm. Data taken at the BALTAZAR facility are show in Figure 2 and Figure S2, Supporting Information. ARPES data obtained at the SGM3 beamline were acquired at photon energies of *h*ν = 49, 53, and 63 eV, with energy and angular resolutions better than 20 meV and 0.1°, respectively. The spot size was 190 µm × 90 µm. Data taken at the SGM3 beamline are shown in Figures 3 and 4; Figures S3 and S4, Supporting Information. All ARPES data were taken at room temperature. Data analysis was performed using Igor Pro (WaveMetrics, Lake Oswego, OR, USA) software package.

## Conflict of Interest

The authors declare no conflict of interest.

## Author Contributions

A.G.C., M.M., C.E.S., M.B., M.H.B, Q.G., and M.D performed ARPES measurements and A.G.C. and M.M. analyzed the data. A.G.C., D.P., Q.G., and M.D. performed AFM measurements. A.G.C., C.E.S., M.M., M.B., and M.D. performed optical imaging. M.B. and D.C. provided experimental support and designed and tested the holding device for the KISS method. A.G.C. and M.D. wrote the manuscript with input and discussion from all co‐authors. M.D. and A.G.C. conceived the project. M.D. provided necessary infrastructure and was responsible for overall project supervision. All authors discussed the results and their interpretation.

## Supporting information

Supporting Information

Supplemental Video 1

## Data Availability

The data used in this study is available on the Zenodo platform at https://doi.org/10.5281/zenodo.7945453.
