# Supplementary material for: In Situ Exfoliation Method of Large‐Area 2D Materials
Source: Adv Sci (Weinh). 2023 May 26;10(22):2301243. doi: 10.1002/advs.202301243 (PMC10401183; doi:10.1002/advs.202301243)
Supplement: Supplementary file 1 — Supporting Information [file ADVS-10-2301243-s002.pdf]

ADVANCED SCIENCE
Open Access

Supporting Information

for *Adv. Sci.*, DOI 10.1002/advs.202301243

In Situ Exfoliation Method of Large-Area 2D Materials

Antonija Grubišić-Čabo, Matteo Michiardi, Charlotte E. Sanders, Marco Bianchi, Davide Curcio, Dibya Phuyal, Magnus H. Berntsen, Qinda Guo and Maciej Dendzik**

Supplementary Information: In situ exfoliation method of large-area 2D materials

Antonija Grubišić-Čabo,*,†,‡ Matteo Michiardi,¶,§ Charlotte E. Sanders,‖ Marco Bianchi,⊥ Davide Curcio,⊥ Dibya Phuyal,‡ Magnus H. Berntsen,‡ Qinda Guo,‡ and Maciej Dendzik*,‡

†*Zernike Institute for Advanced Materials, University of Groningen, 9747 AG Groningen, The Netherlands*

‡ *Department of Applied Physics, KTH Royal Institute of Technology, Hannes Alfvéns väg 12, 114 19 Stockholm, Sweden*

¶*Quantum Matter Institute, University of British Columbia, Vancouver, BC V6T 1Z4, Canada*

§*Department of Physics and Astronomy, University of British Columbia, Vancouver, BC V6T 1Z1, Canada*

‖*Central Laser Facility, STFC Rutherford Appleton Laboratory, Harwell 0X11 0QX, United Kingdom*

⊥*School of Physics and Astronomy, Aarhus University, 8000 C Aarhus, Denmark*

E-mail: a.grubisic-cabo@rug.nl; dendzik@kth.se

Characterization of WSe_2 on Au(111) substrate

WSe_2 was exfoliated onto an Au(111) single crystal using the KISS method. Optical microscopy and atomic force microscopy (AFM) data for this single-layer WSe_2 flake was shown in Figure 1 of the main text. Further characterization done on this flake is shown in supplementary Figure S1, while a large-scale AFM image for the same WSe_2/Au(111) is shown in supplementary Figure S2. Low-Energy Electron Diffraction (LEED) data, Figure S1**a**, confirm the presence of only one WSe_2 domain, which is rotated 13° relative to the underlying Au(111) substrate. Angle-Resolved Photoemission Spectroscopy (ARPES) data taken around the high-symmetry point $\bar{\Gamma}$, Figure S1**b** (left), show the presence of WSe_2 states and Au surface state. The sample is a mixture of a single layer (SL) with a bilayer (BL) contribution, as indicated by the presence of two WSe_2 bands at $\bar{\Gamma}$. ARPES data taken around the $\bar{K}$ point, Figure S1**b** (right), show the spin-split valence band (VB) of WSe_2, with a splitting of approximately 440 meV. Bands arising from Au *sp* orbitals are also visible and intersect with the WSe_2 VB states.

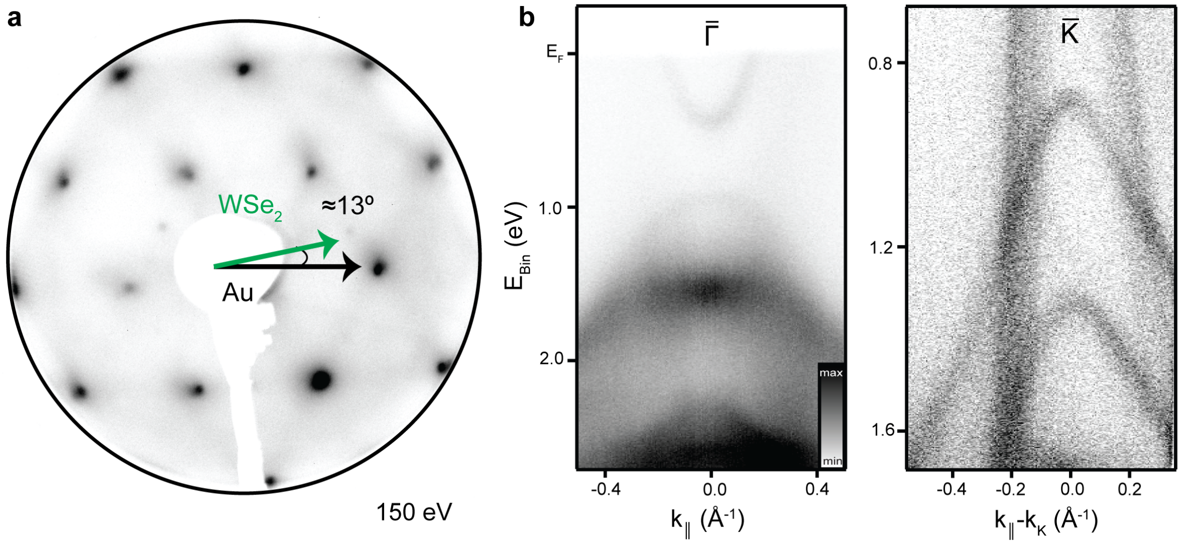

Figure S 1: WSe_2 on Au(111). **a** LEED image showing single crystal WSe_2 rotated 13° with respect to the underlying Au(111). The black arrow indicates Au, while the green arrow indicates the WSe_2 diffraction spot. **b** ARPES data of $\bar{\Gamma}$ (left) and $\bar{K}$ point (right). The sample is a single layer (SL) with a bilayer (BL) contribution.

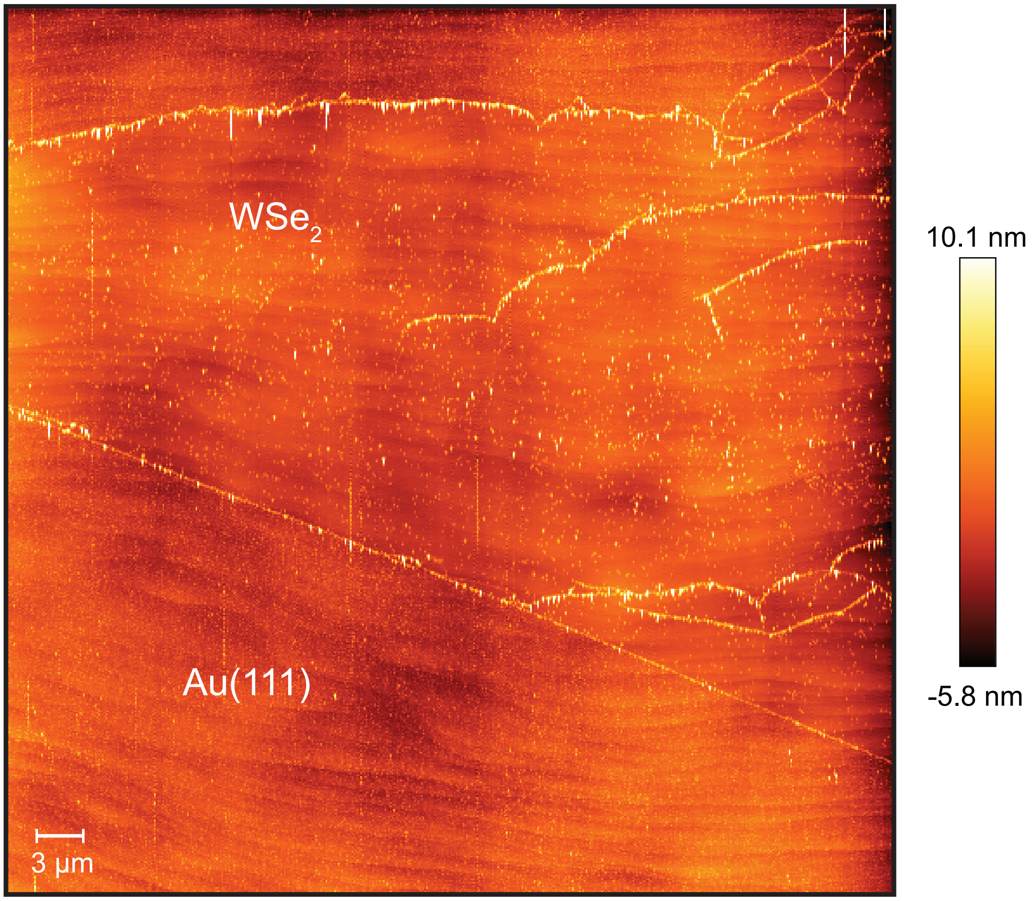

Figure S 2: Large scale AFM image of WSe_2 on Au(111).

Monolayer-bilayer mix of MoS_2 on Ag(111) substrate

MoS_2 was exfoliated by the KISS method onto Ag(111), see Figure S3. Unlike other samples shown in the main text and supplementary information, this sample consists predominantly of BL regions. Optical microscopy, Figure S3**a**, shows that this sample is a mix of SL and BL, with smaller bulk regions present at the edges of some flakes. ARPES data, Figure S3**b**, show silver states (SS, *sp*-bands) coexisting with the MoS_2 bands. The VB maximum (VBM) for this sample is located at the $\bar{\Gamma}$ point, as expected for a BL. The spin-splitting of states at $\bar{K}$ is approximately 150 meV, as expected for MoS_2 on Ag.

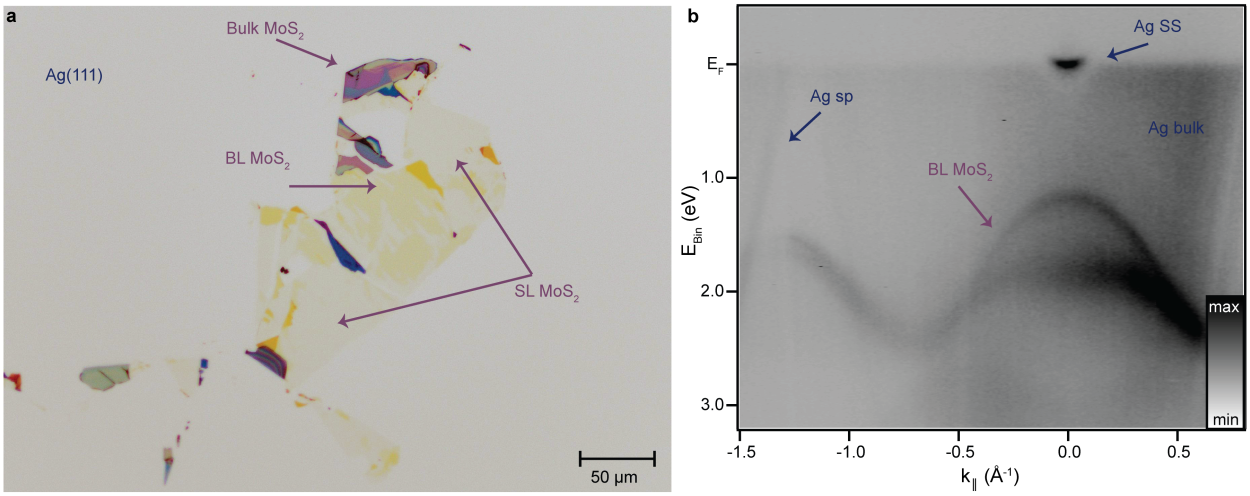

Figure S 3: Bilayer MoS_2 on Ag(111). **a** Optical microscopy image with SL, BL and bulk MoS_2 regions indicated. **b** ARPES data showing MoS_2 along the $\bar{\Gamma}$-$\bar{K}$ direction. Two bands are visible at the $\bar{\Gamma}$ point, confirming that the sample is predominantly a BL.

Fermi surface of WTe_2 on Ag(111) substrate

KISS-exfoliated flakes of WTe_2 on Ag(111) show 2D and bulk-like (3D) character in its energy dispersion. However, both regions show only a single domain, as seen in the Fermi surface (FS) maps of both regions. Figure S4 shows FS maps of WTe_2 on Ag(111), focusing on 2D (S4**a**) and bulk (S4**b**) regions. In both cases, only a single FS is visible, confirming that the exfoliated WTe_2 flake is single-crystal and has only one domain.

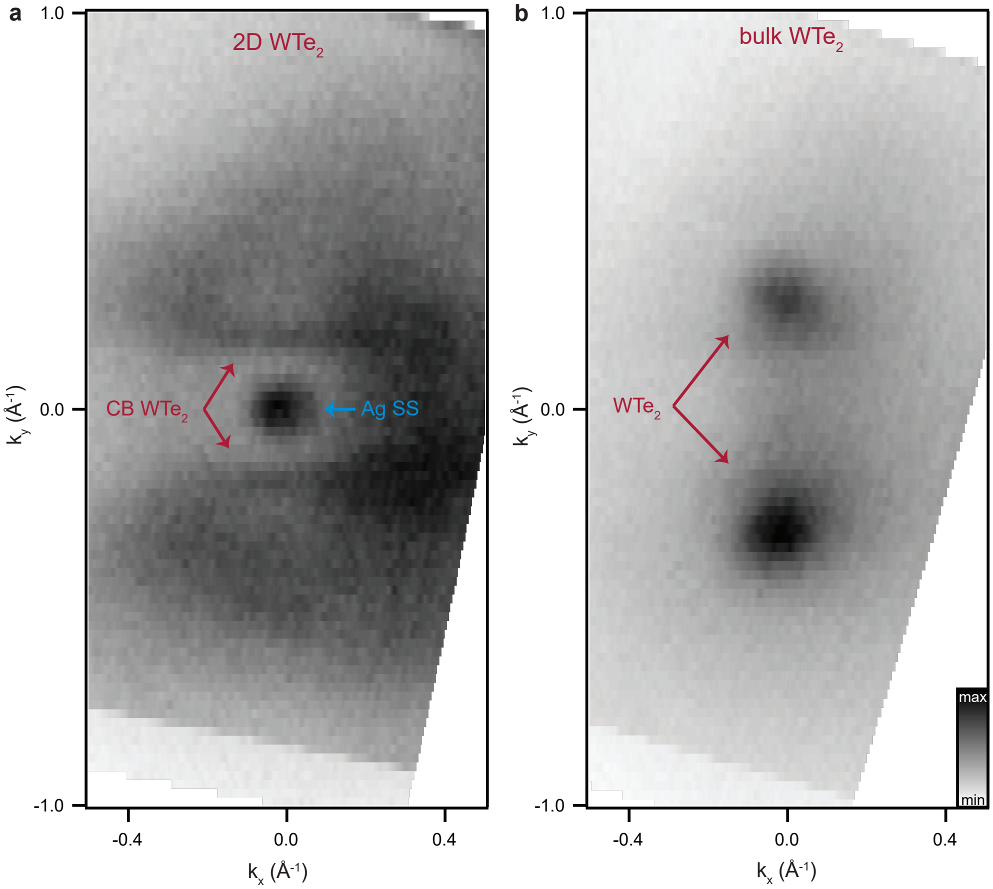

Figure S 4: Fermi surface of WTe_2 on Ag(111). **a** 2D WTe_2 with WTe_2 conduction band (CB) and Ag surface state (SS) indicated with arrows. **b** Bulk-like WTe_2, WTe_2 states are indicated by arrows. In both cases, the Fermi surface shows only a single domain.

Spatial mapping in the valence band region of MoS_2 on Ag(111)

KISS-exfoliated flakes were located on the substrate by ARPES raster-mapping focusing on the signal originating from core-levels or valence bands. An example of such a map is shown in Figure S5 for MoS_2 on Ag(111). Figure S5**a** shows a real-space map of the sample, with measurement locations for SL and BL MoS_2, clean Ag, and the Ta sample holder indicated by colored stars. Figure S5**b** shows SL MoS_2, and Figure S5**c** shows BL MoS_2 states at $\bar{\Gamma}$. The data was recorded on 2 different flakes, as seen in Figure S5**a**. Figure S5**d** shows clean Ag substrate, where no MoS_2 is exfoliated, while Figure S5**e** shows states coming from the Ta sample holder on which the Ag(111) substrate was mounted. The intensity in Figure S5**a** stems from the binding energy region marked with a black square in Figure S5**c**, where a SL MoS_2 band is expected.

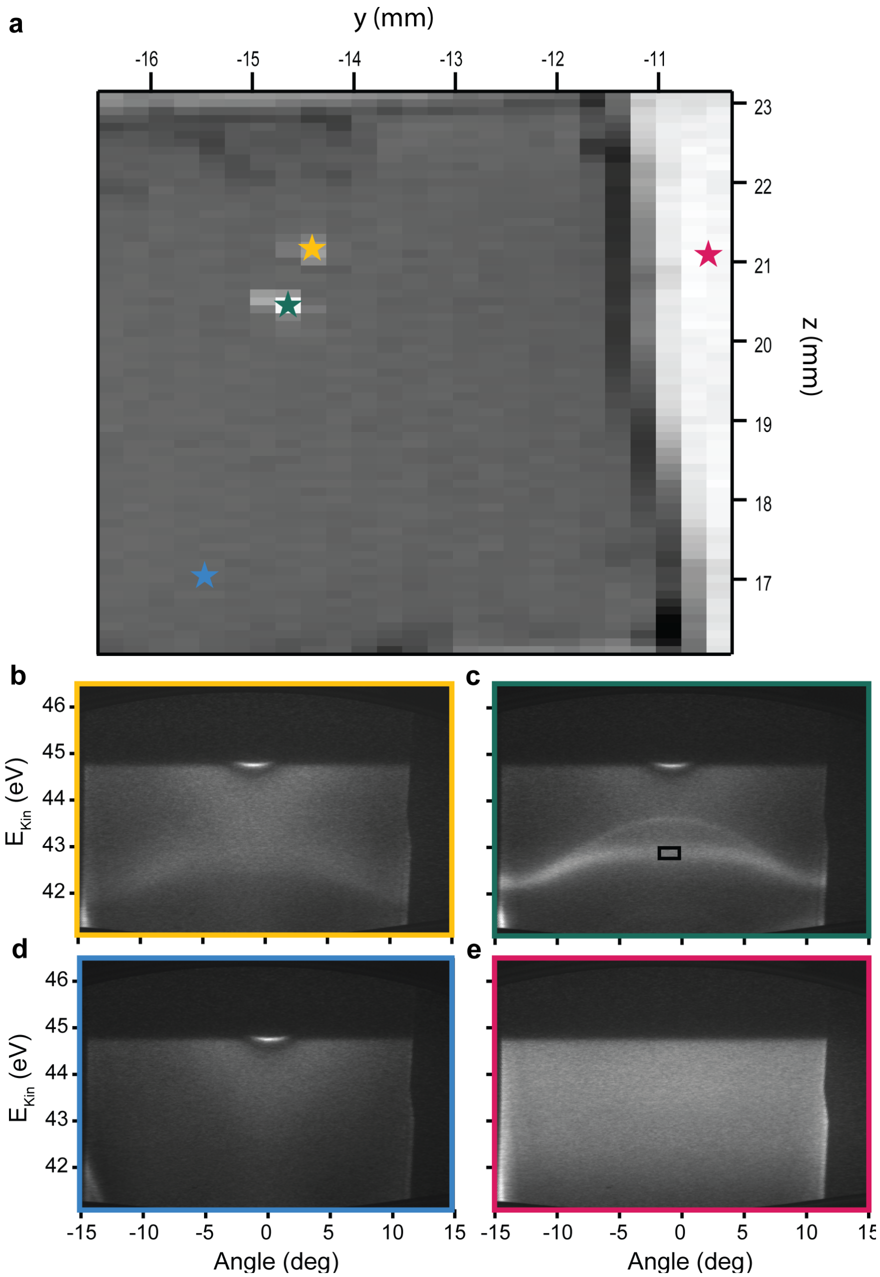

Figure S 5: An example of a spatial map used to locate KISS-exfoliated MoS_2 on Ag(111). **a** Spatial map showing 2 flakes of MoS_2 on the Ag(111). The colored stars mark the locations where the band structures in **b-e** were measured. **b** SL MoS_2/Ag(111), **c** BL MoS_2/Ag(111), **d** Ag surface state, with no MoS_2 states visible and **e** Ta sample plate. The frame colors match the stars indicating the location on the spatial map where the data is recorded. The black square in **c** shows the region from which the intensity for the real space map in **a** is captured.

Sample holders used for *in situ* exfoliation

Examplary sample holders used for *in situ* exfoliation are shown in figure S6. While the majority of the *in situ* exfoliation was done with spring-loaded holders (Figure S6 **b,c**), it is also possible to do with holders without a spring (Figure S6 **a**). Particular design differs based on the holders in use at the particular system, but it is generally very adaptable. The springs that were used in the experiments had the maximal load of 2.57 N, and the force during the KISS-exfoliation is measured to be 2.45 N.

a **b** **c**

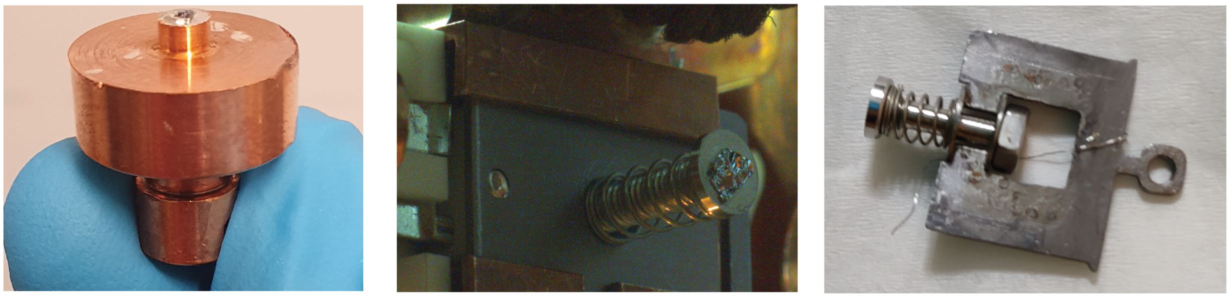

Figure S 6: Photographs of various sample plates used in KISS-exfoliation. Design differs slightly depending on the type of sample plate that is in use. **a** Sample plate used at the BALTAZAR facility without a loaded spring. Spring-loaded sample plate adapted for **b** home built sample holder of the SGM3 beamline and **c** for flag-type sample holder.

AFM analysis of substrates and 2D materials morphology

We have performed AFM measurements on three different types of metallic substrates used in this work, Figure S7, and acquired optical images of typical surfaces obtained on layered materials by UHV-cleaving, Figure S8, in order to determine the role of substrate and crystal surface flatness on the UHV exfoliation success. Surface roughness analysis shows that Au(111)/mica has a more rough surface, average roughness 2.7 nm, compared to the Au(111) single crystal (1.8 nm) and Ag(111)/mica (1.4 nm). However, it is more likely that the uneven surface of UHV-cleaved crystals, clearly seen in the Figure S8, has much higher role on the success of the UHV-exfoliation, and size of the obtained flakes, as it will have higher influence on the contact quality.

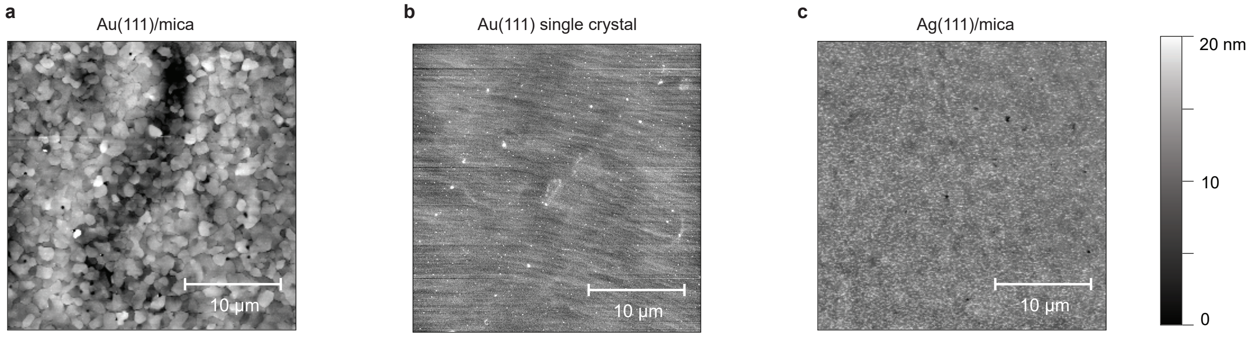

Figure S 7: In-air AFM images of UHV-cleaned substrates: Au(111)/mica, Au(111), Ag(111)/mica in **a-c**. The average roughness is 2.7 nm, 1.8 nm and 1.4 nm, respectively.

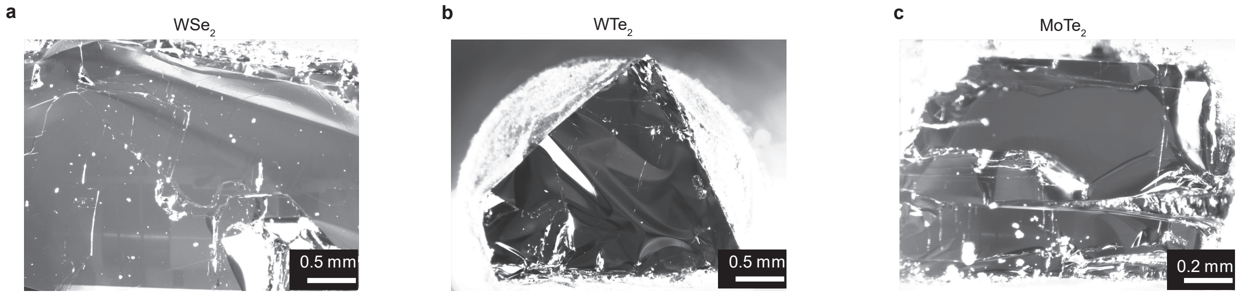

Figure S 8: Optical images of UHV-cleaved 2D materials: WSe_2, WTe_2, $MoTe_2$ in **a-c**.

Large scale optical images of samples prepared with *in situ* exfoliation

Large scale images for UHV exfoliated flakes shown in the main text are shown in Figure S9, Majority of the exfoliated flakes are very thin, with thicker regions typically at the flake edges.

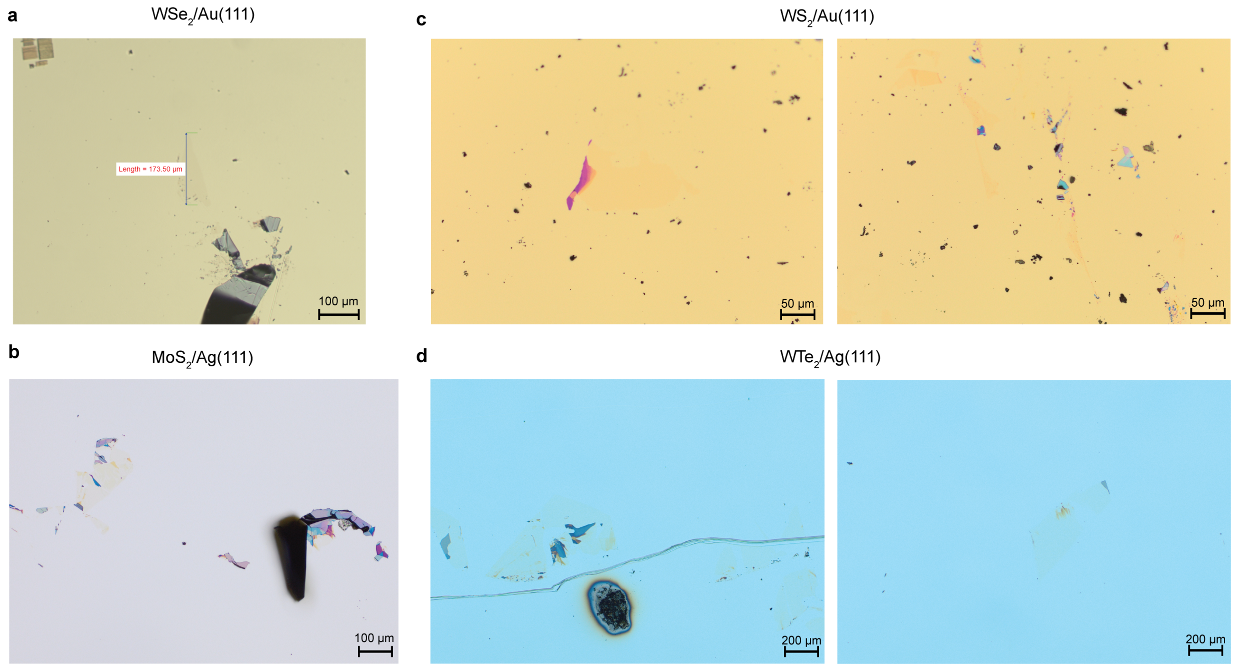

Figure S 9: Larger scale images for data presented in the main text. **a** WSe_2/Au(111) single crystal, **b** MoS_2/Ag(111)/mica, **c** Two different spots of WS_2/Au(111)/mica and **d** Two different spots of WTe_2/Ag(111)/mica.
